# Clinical Significance of Albumin- and Bilirubin-Based Biomarkers in Glaucoma: A Retrospective Case-Control Study

**DOI:** 10.1155/2022/8063651

**Published:** 2022-03-04

**Authors:** Chong He, Gao Zhang, Jing Fu, Rui Zhang, An Li, Donghua Liu, Binghong Li, Yang Chen, Bolin Deng, Yilian Chen, Ping Shuai, Fang Lu

**Affiliations:** ^1^Clinical Immunology Translational Medicine Key Laboratory of Sichuan Province, Sichuan Provincial People's Hospital, University of Electronic Science and Technology of China, Chengdu, China; ^2^Medico-Engineering Cooperation on Applied Medicine Research Center, University of Electronic Science and Technology of China, Chengdu, China; ^3^Health Management Center, Sichuan Provincial People's Hospital, University of Electronic Science and Technology of China, Chengdu, China; ^4^Department of Ophthalmology, Sichuan Provincial People's Hospital, University of Electronic Science and Technology of China, Chengdu, China

## Abstract

Glaucoma is the second leading cause of global blindness. The etiology of glaucoma is complicated. In addition to elevated intraocular pressure (IOP), several other mechanisms have been implicated in pathogenesis, such as oxidative stress and systemic inflammation. Serum albumin (ALB) and bilirubin (BIL) have been reported to have potent antioxidant properties and contribute to maintain redox homeostasis in various diseases. However, associations between these parameters and glaucoma remain mostly unknown. Here, we conducted a retrospective case-control study, revealing that serum ALB, total BIL (TBIL), and indirect BIL (IBIL) levels were markedly lower in glaucoma patients than those in healthy controls. Furthermore, the neutrophil-to-ALB (NAR), neutrophil-to-TBIL (NTBR), and neutrophil-to-IBIL (NIBR) ratios were greatly higher in glaucoma. Additionally, interestingly, lower ALB and BIL levels and higher NAR, NTBR, and NIBR were associated with severer glaucomatous visual impairment, and NAR, NTBR, and NIBR showed good accuracy as diagnostic tests for glaucoma severity, suggesting these indices might be useful as discriminative biomarkers for disease severity. Our current findings demonstrate associations between ALB, BIL, NAR, NTBR, NIBL, and glaucoma. It might be useful to use NAR, NTBR, and NIBR as predictive markers for disease severity and employ ALB/BIL as alternative therapy or adjuvant medicines in glaucoma patients.

## 1. Introduction

Glaucoma is a class of neurodegenerative disorders with a complicated etiology that primarily damages the retinal ganglion cells (RGC) and optic nerve (ON), eventually resulting in permanent vision loss [1]. The existing poor situation with early detection and prevention against glaucoma has spurred doctors and researchers to seek novel remedies by employing smart biotechnological techniques. The most difficult challenges are disease-specific changes in the molecular basis and tissue specificity of molecular patterns. Increasing studies using animal models for glaucoma give significant information about impacted biochemical pathways and possible targets in this disease, but they are sometimes not suitable for noninvasive examinations on patients [2].

Although elevated intraocular pressure (IOP) is the most major risk, it remains to be elucidative regarding the exact mechanism of glaucomatous damage. Significantly, other factors including vascular factors [3], oxidative stress [4], and neuroinflammation [5] have been implicated as underlying cellular and molecular mechanisms. Oxidative stress has been considered as a major etiologic factor in glaucoma pathogenesis [6]. It has been reported that there is an imbalance between prooxidative state and antioxidant defense activity in glaucoma patients [7]. Aqueous humor of glaucoma patients exhibited a considerable increase in antioxidant enzyme activity, such as superoxide dismutase. While nonenzymatic antioxidants including vitamins C and E levels in the aqueous humor were consistently reduced compared to cataract controls [8], glaucoma patients were found to have lower systemic antioxidant capacity such as biologic antioxidant potential (BAP) [9]. Changes of crucial mediators of oxygen homeostasis in the glaucoma aqueous humor proteome suggested a role of oxidative stress in the glaucomatous neuronal injury, indicative of a potential application for detection of these mediators in prediction of disease progression [10].

Serum albumin (ALB) and bilirubin (BIL) are conventionally two indicators of hepatobiliary function in clinical biochemical tests, which are routine hospital examinations in clinical practice. Of note, both of them have been reported to have powerful antioxidant properties and could serve as serum biomarkers in oxidative stress/inflammation-associated disorders [11]. In the context of oxidative stress, ALB is transformed into an oxidized status, which is undetected by common laboratory methods, leading to reduced serum ALB levels in patients [12]. On the other hand, neurotoxicity and oxidative stress can be caused by high levels of BIL, but molecular biology investigations have shown that bilirubin itself is a powerful antioxidant. The levels of both serum ALB and indirect BIL (IBIL) have been observed to be significantly lower in neurodegenerative diseases [13–18]. However, the association between serum ALB or BIL and glaucoma remains largely unknown.

In this retrospective case-control study, we aimed to compare serum ALB and BIL levels between the control group and glaucoma patients. Additionally, we sought to combine ALB/BIL levels with parameters for systemic inflammation such as blood neutrophil counts as neutrophil-to-ALB (NAR), neutrophil-to-total BIL (NTBR), and neutrophil-to-IBIL (NIBR) ratios and explore the relationships between these indices and glaucoma severity.

## 2. Materials and Methods

### 2.1. Subjects

In the current study, we recruited 175 patients were recruited from Sichuan Provincial People's Hospital (Chengdu, China). Our study was conducted in accordance with the Declaration of Helsinki and approved by the Institutional Review Board for Clinical Research of Sichuan Provincial People's Hospital (no. 201968). All subjects were well informed about the study and potential risk and signed an informed consent before participation. Demographics of patients are shown in Table 1. As reported previously [19], glaucoma was diagnosed by ophthalmologists according to ophthalmic examinations in combination with age, family history, and glaucomatous clinical manifestation. Following ophthalmic examinations were performed: anterior chamber angle assessment, IOP (≥3 measurements and the average was recorded), cup-to-disk ratio, visual field loss, and RNFL thickness. The selection of patients with glaucoma was conducted as described previously [19]. Inclusion criteria: no secondary glaucoma or other visual disorders, no intraocular surgery in the last 6 months, no hematopoietic system disease, no hepatobiliary disease, no coagulation abnormalities, without taking medications that can affect blood cell components or serum biochemistry profiles, no any systemic diseases (such as hypertension, diabetes, infections, systemic autoimmune diseases, and cancers), or no other neurodegenerative disorders (such as Parkinson's disease and Alzheimer's disease). The severity of glaucoma was determined based on the mean deviation (MD) of visual field: early indicates visual field MD of greater than -6 dB; moderate, -12 dB to -6 dB; and severe, no greater than -12 dB [19, 20].

Healthy controls, who participated in yearly health screenings during the study period and had no clinical evidence of glaucoma or a family history of glaucoma, were also consecutively enrolled from Sichuan Provincial People's Hospital. Exclusion criteria of healthy controls: subjects with complaints of eye discomfort, IOP elevation (≥21 mmHg), any recent surgeries, any hematopoietic system disorders, any hepatobiliary diseases, any coagulation abnormalities, taking medications that can affect blood cell components or serum biochemistry profiles, any systemic diseases (such as hypertension, diabetes, infections, systemic autoimmune diseases, and cancers), or any other neurodegenerative disorders. According to the inclusion and exclusion criteria, a total of 293 age- and gender-matched healthy controls were included.

### 2.2. Collection and Analysis of Blood Samples

Blood cell counts and serum biochemical tests were performed when patients were diagnosed. Peripheral venous blood was collected using EDTA-anticoagulated Vacutainer CPT tubes (BD Biosciences, San Diego, CA, USA). Blood sera were collected using serum separator tube (BD Biosciences). At the time of blood sampling, no subjects had an acute infection or were taking any medication known to influence blood cell components or serum biochemistry profiles. Blood and serum samples were sent to the laboratory and processed within 1 hour. Blood cell counts were required on a hematology analyzer (Abbott CELL DYN 3700 System, Ramsey, Minnesota 55303, USA). Serum total protein (TP), ALB, globulin (GLB), TBIL, DBIL, and IBIL were analyzed on an Architect C 16000 (Abbott) device at the biochemistry laboratory of Sichuan Provincial People's Hospital. The NAR, NTBR, and NIBR were calculated as the ratio of neutrophil counts (×10^9^/L)-to-ALB (g/L), neutrophil counts (×10^9^/L)-to-TBIL (*μ*mol/L), and neutrophil counts (×10^9^/L)-to-IBIL(*μ*mol/L), respectively.

### 2.3. Statistical Analysis

The statistical analysis was performed using a Prism software Version 8.4 (Graphpad Software, San Diego, California, USA). Kolmogorov-Smirnov test was performed for checking data normality. For nonnormally distributed data, the differences between two groups were examined using Mann–Whitney test. When there were ≥3 groups, the Kruskal-Wallis test was performed to assess the difference among groups, followed by the Dunn's multiple comparisons test to determine the differences of each group with every other group. For normally distributed data, unpaired Student's *t* test (two-tailed) was used for comparison between 2 groups. *p* < 0.05 was considered statistically significant. Receiver operating characteristics (ROC) curve analysis was performed, and the area under the ROC curve (AUC) was calculated to determine the diagnostic abilities of each variable in indicated scenarios. Youden index was calculated as (sensitivity + specificity‐1) to measure the diagnostic accuracy of each variable.

## 3. Results

### 3.1. Demographics of the Participants

As shown in Table 1, we enrolled 175 patients with glaucoma in this study, including 105 primary angle-closure glaucoma (PACG) and 70 primary open-angle glaucoma (POAG). Age- and gender-matched control participants (*n* = 293) were also enrolled. The IOP of enrolled glaucoma patients was 25.1 ± 11.25 mmHg. Patients were divided into 3 groups stratified according to glaucomatous damage severity based on the mean deviation (MD) of visual field (early, MD: greater than -6 dB, *n* = 41; moderate, MD: -12 dB to -6 dB, *n* = 65; severe, MD: no greater than -12 dB, *n* = 69). The age, gender, and IOP did not differ between the PACG and POAG groups. If both eyes of the same individual were affected by glaucoma, only one eye was randomly selected.

### 3.2. Serum ALB and BIL Levels Are Significantly Lower in Glaucoma Patients

As demonstrated in Table 1, serum ALB (43.5, 42.1 45.3 g/L) and TP (71.9, 68.6 74.7 g/L) levels of glaucoma patients were significantly lower than those in the control group (ALB, 44.6, 43.1 46.6 g/L, *p* < 0.0001; TP, 72.5, 70.0 75.6 g/L, *p* = 0.0087). However, the GLB level did not show any evident differences. Furthermore, glaucoma patients had significantly lower levels of both serum TBIL and IBIL (15.4, 12.3 19.2 mol/L and 10.2, 7.9 12.7 mol/L, respectively) than control participants (17.2, 13.9 20.7 mol/L, *p* = 0.0052 and 11.8, 9.5 14.9 mol/L, *p* < 0.0001, respectively). The DBIL level was comparable between patients and controls.

### 3.3. NAR, NTBR, and NIBR Are Remarkably Higher in Patients with Glaucoma

In addition, we compared a newly identified index calculated as the ratio of neutrophil counts over serum ALB levels (NAR) between glaucoma patients and control participants and found glaucoma patients had a remarkably higher NAR (0.092, 0.075 0.116) than the control group (0.076, 0.060 0.094, *p* < 0.0001) (Table 1). Next, we moved forward to determine whether the neutrophil-to-BIL ratio also altered in glaucoma. As shown in Table 1, both NTBR and NIBR were expectedly higher in patients than those in controls (0.247, 0.197 0.354 to 0.204, 0.149 0.267, *p* < 0.0001 and 0.382, 0.280 0.552 to 0.283, 0.207 0.391, *p* < 0.0001, respectively).

Furthermore, we compared abovementioned indices (serum ALB, TBIL, and IBIL levels, and NAR, NTBR, and NIBR) between patients with PACG and POAG. None of them were significantly different (Table 2).

### 3.4. Associations of Serum ALB, BIL, NAR, NTBR, and NIBR with disease severity

**Figure 1:**
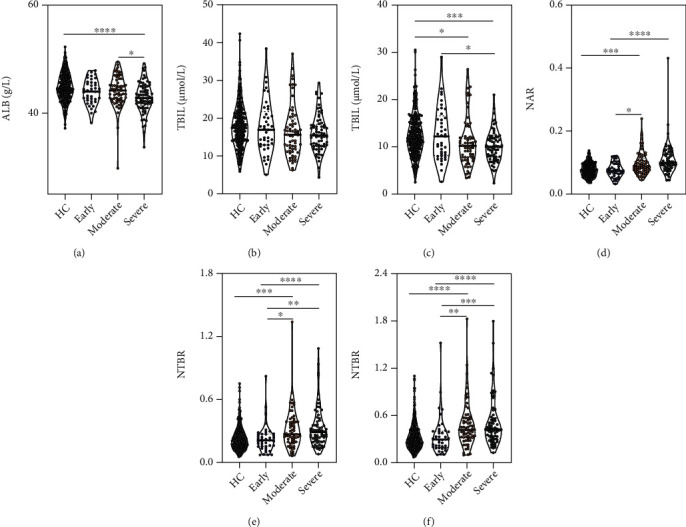
Associations of serum ALB, BIL, NAR, NTBR, and NIBR with disease severity. Serum levels of (a) ALB, (b) TBIL, (c) IBIL, (d) NAR, (e) NTBR, and (f) NIBR among different glaucoma patient groups stratified according to disease severity. The severity of glaucoma was determined based on the mean deviation (MD) of visual field: early (*n* = 41) indicates visual field MD of greater than -6 dB; moderate (*n* = 65), -12 dB to -6 dB; and severe (*n* = 69), no greater than -12 dB. Kolmogorov-Smirnov test was performed for checking data normality. The differences of all parameters among three groups were first examined by Kruskal-Wallis test, followed by Dunn's multiple comparisons test to determine the differences of each group with every other group (adjusted *p* values are shown). ^∗^*p* < 0.05, ^∗∗^*p* < 0.01, ^∗∗∗^*p* < 0.001, and ^∗∗∗∗^*p* < 0.0001. HC: healthy controls; ALB: albumin; TBIL: total bilirubin; IBIL: indirect bilirubin; NAR: neutrophil-to-albumin ratio; NTBR: neutrophil-to-total bilirubin ratio; NIBR: neutrophil-to-indirect bilirubin ratio.

Next, we aimed to compared ALB, TBIL, IBIL, NAR, NTBR, and NIBR among different glaucoma patient groups stratified according to disease severity. As shown in Figure 1, the age was closely matched among three glaucoma groups. The ALB level was the lowest in the severe group, while it was comparable between the early and moderate groups. Similarly, the TBIL or IBIL level was the lowest in the severe group, followed by the moderate group, and then the early group, but only the IBIL level between the early and severe groups showed statistical significance. As for NAR, NTBR, and NIBR, all three indexes were upward with increasing disease severity, and statistical significances were observed when compared the early group to the moderate and severe groups, respectively.

### 3.5. Receiver Operating Characteristic (ROC) Curve Analysis

We evaluated the discriminative ability of ALB, TBIL, IBIL, NAR, NTBR, and NIBR to differentiate glaucoma patients from the control group. As shown in Table 3, ALB (AUC = 0.6253, *p* < 0.0001), IBIL (AUC = 0.6105, *p* < 0.0001), NAR (AUC = 0.6673, *p* < 0.0001), NTBR (AUC = 0.6338, *p* < 0.0001), and NIBR (AUC = 0.6566, *p* < 0.0001) showed sufficient accuracy as diagnostic tests between glaucoma patients with controls. However, the Youden index maximal of all variables was below 0.3. In addition, we questioned whether the variables could be discriminative markers among glaucoma patients with different disease severity. Based on the AUC and Youden index, we found that NAR (AUC = 0.7554, Max.Youden = 0.46), NTBR (AUC = 0.7027, Max.Youden = 0.36), and NIBR (AUC = 0.7250, Max.Youden = 0.40) showed good accuracy to discriminate patients with severe glaucoma from those with early glaucoma (Table 4), suggesting that these 3 indexes might be useful to help discriminate the disease severity.

## 4. Discussion

In the current study, we analyzed the alterations of serum ALB and BIL in combination with blood neutrophil counts, revealing that (1) serum ALB, TBIL, and IBIL were significantly lower, while NAR, NTBR, and NIBR were significantly higher in patients with glaucoma than those in healthy controls. (2) Serum ALB and IBIL levels were negatively, while NAR, NTBR, and NIBR were positively associated with the clinical severity of visual impairment in glaucoma patients; (3) NAR, NTBR, and NIBR showed good accuracy as diagnostic tests for glaucoma severity. These observations prompt us to speculate their potential to serve as biomarkers to monitor the disease progression.

Although the pathogenesis of glaucoma is complex and mostly unclear, the role of disturbed oxidative/antioxidative status has been emphasized in patients and animal models. Oxidative stress is a pathological condition induced by an excess of reactive oxygen species (ROS) at levels greater than the body's antioxidant capability. This may result in cell death and the buildup of apoptotic residues, as well as the development of autoantibodies and autoimmune cascade response activation. ALB is usually used to reflect the nutritional status and the ability of the liver to synthesize protein. ALB also plays as an essential antioxidant in plasma, where continuous oxidative stress exposure occurs. ALB can sponge various types of molecules as a “tramp steamer” in the circulation, including transition metals (e.g., copper and iron) and polyunsaturated fatty acids, both of which are powerful ROS-generator after reacting with oxygen [21]. Thus, its ligand-binding abilities lead to many antioxidant activities. Even though impairments of ALB molecule as well as its antioxidant properties have been thought as “biologically insignificant” due to its large amount in the serum and its rapid clearance from the circulation [22, 23], evidences showed that impaired antioxidant capacities of ALB may be associated with many pathological conditions, such as diabetes and chronic kidney diseases [24, 25]. In addition, oxidized ALB has been suggested as an oxidative stress marker in such diseases as Alzheimer and Parkinson's disease [26, 27]. Efforts have been made to investigate the redox state ALB in patients with glaucoma [28–31]. For example, the redox state of vitreous ALB has been suggested as a biomarker for the redox situation in the vitreous of patients with POAG [30]. Ischemia-modified albumin levels are significantly higher in patients with PACG, indicating a role as a biomarker available for assessing oxidative stress in the disease [28]. According to these evidences, we here explored serum ALB levels in patients with glaucoma and revealed lower ALB levels in glaucoma than those in healthy controls.

Although BIL is conventionally considered to be an end product of heme degradation and it is cytotoxic at high concentrations, recent evidences have discovered a variety of biological properties of this the metabolite of iron porphyrin at physiological concentrations, such as antioxidative, anti-inflammatory, and neuroprotective [32]. BIL could play as an antioxidant via ROS scavenging and NADPH oxidase activity inhibiting mechanisms, leading to a decrease in oxidative stress. Existing evidences have revealed reduced levels of serum BIL in patients with neurodegenerative or neuroinflammatory disorders such as multiple sclerosis and Parkinson's disease [33]. Furthermore, clinical investigations have linked serum BIL levels to the risk of cardiovascular disorders such as coronary artery disease and peripheral artery disease [34, 35]. In clinical settings, the relationships of BIL with endothelial function in vascular disorders have been studied. In spite of accumulating evidences showing both oxidative stress and vascular factors are tightly involved in glaucoma pathogenesis, few studies have paid attention to BIL in glaucoma. Our findings here showed lower levels of TBIL and IBIL in glaucoma patients than those in healthy controls.

Evidences suggest that not only local neuroinflammation in the retina but also systemic inflammation plays a critical role in the pathogenesis of glaucoma [36]. As a consequence, systemic inflammatory indices have been proposed as indicators for disease diagnosis and clinical outcome prediction. Therefore, methods based on peripheral venous blood, which is inexpensive, simple, commonly available, and adequate for cellular and molecular analysis in small amounts (even a few milliliters), may have practicability in screening and diagnosis for high-risk people. Blood indicators such as white blood cells (WBC), neutrophils, neutrophil-to-lymphocyte ratio (NLR), platelet-to-lymphocyte ratio (PLR), and lymphocyte-to-monocyte ratio (LMR) are straightforward for systemic inflammation. Such biomarkers have shown potent values as novel tools for the early identification and individual screening of glaucoma. WBC and neutrophil counts, as well as NLR and PLR, are found to be significantly increased, while lymphocyte counts, platelets, and LMR are decreased in patients with PACG. NLR exhibits the potential value to reflect the disease severity [36–39]. Recently, informative parameters combining blood cells and serum biochemical indices such as NAR emerged to indicate systemic inflammation and have been applied in inflammatory and vascular diseases and cancers. In patients with cardiogenic shock (CS), high levels of NAR are linked to growing risk of mortality caused by this lethal clinical emergency. Notably, the diagnostic sensitivity of NAR is greater than blood neutrophil or serum albumin level alone [40]. Varim et al. conducted a retrospective study including 144 patients with COVID-19 and found a positive relationship between NAR and poor prognosis and mortality in patients with a COVID-19 infection [41]. Moreover, NAR has been also demonstrated to be a positive risk factor for delayed cerebral ischemia after aneurysmal subarachnoid hemorrhage [42]. To our best knowledge, this is the first report exploring the association between NAR and glaucoma. Here, we showed an evident increase of NAR in glaucoma patients compared to that in the control group, which indicated the disease severity. Moreover, both NTBR and NIBR are positively correlated with the visual impairment of glaucoma patients. In addition, NAR, NTBR, and NIBR showed good accuracy as diagnostic tests for glaucoma severity, suggesting that they might be promising biomarkers to monitor the disease progression. As far as we know, little information about the NBIR in clinical practice. It will be interesting to look into the clinical significance of this index in different diseases in future studies.

We do realize some limitations in the current study: (1) although we showed potential value of ALB, BIL, NAR, and NBR to discriminate glaucoma from healthy controls, the diagnostic accuracy of these tests needs to be improved in following studies with larger sample sizes; (2) the current study is a cross-sectional case-control study, in which we were not able to investigate the potential mechanisms underlying the associations between ALB/BIL and glaucoma. Therefore, further longitudinal studies are required; (3) due to the study design, we showed the associations between these biomarkers and glaucoma severity, instead of disease progression. Follow-up studies are required to examine the application value of them to predict glaucoma progression.

To sum up, our current findings demonstrate associations between ALB, BIL, NAR, NTBR, NIBL, and glaucoma. It might be useful to use NAR, NTBR, and NIBR as predictive markers for disease severity and employ ALB/BIL as an alternative therapy or adjuvant medicines in glaucoma patients.

## Figures and Tables

**Table 1:** Demographics and parameters of glaucoma patients and healthy controls.

	Glaucoma	Healthy controls	*p* value
*n*	175	293	—
Age (year)	62 (54 71)	63 (54 71)	0.0838
Gender			
Female	94	136	0.1517
Male	81	157	
PACG/POAG	105/70	—	
IOP (mmHg)	21.1 (16.8 32.5) ↑	15.8 (12.5 17.8)	<0.0001
Glaucoma severity			
Early	32	—	—
Moderate	38	—	—
Severe	105	—	—
NEU (∗10^9^/L)	4.0 (3.3 5.0) ↑	3.4 (2.7 4.1)	<0.0001
TP (g/L)	71.9 (68.6 74.7) ↓	72.5 (70.0 75.6)	0.0087
ALB (g/L)	43.5 (42.1 45.3) ↓	44.6 (43.1 46.6)	<0.0001
GLB (g/L)	28.21 ± 3.76	28.22 ± 3.55	0.9770
TBIL (mol/L)	15.4 (12.3 19.2) ↓	17.2 (13.9 20.7)	0.0052
DBIL (mol/L)	5.2 (4.1 6.5)	4.9 (4.2 5.9)	0.0925
IBIL (mol/L)	10.2 (7.9 12.7) ↓	11.8 (9.5 14.9)	<0.0001
NAR	0.092 (0.075 0.116) ↑	0.076 (0.060 0.094)	<0.0001
NTBR	0.247 (0.197 0.354) ↑	0.204 (0.149 0.267)	<0.0001
NIBR	0.382 (0.280 0.552) ↑	0.283 (0.207 0.391)	<0.0001

Data are presented as median (IQR) except for GLB, which is presented as mean ± SD. Abbreviations: IQR: interquartile range; PACG: primary angle-closure glaucoma; POAG: primary open-angle glaucoma; IOP: intraocular pressure; NEU: neutrophil counts; TP: total protein; ALB: albumin; GLB: globulin; TBIL: total bilirubin; DBIL: direct bilirubin; IBIL: indirect bilirubin; NAR: neutrophil-to-albumin ratio; NTBR: neutrophil-to-total bilirubin ratio; NIBR: neutrophil-to-indirect bilirubin ratio. Kolmogorov-Smirnov test was performed for checking data normality. The differences of all parameters between glaucoma patients and healthy controls were examined by Mann–Whitney test, except for the gender and GLB, which was examined by Chi-square test and unpaired Student's *t* test (two-tailed), respectively. *p* < 0.05 was considered statistically significant.

**Table 2:** Demographics and parameters of glaucoma patients with PACG and POAG.

	PACG	POAG	*p* value
n	105	70	—
Age, y	64 (55 70)	60 (54 72)	0.8523
Glaucoma severity			
Early	22	19	0.6172
Moderate	41	24	
Severe	42	27	
NEU (∗10^9^/L)	4.1 (3.4 5.1)	3.7 (3.1 4.7)	0.1490
TP (g/L)	71.2 (67.7 74.4)	72.5 (69.9 74.8)	0.0845
ALB (g/L)	43.4 (42.1 45.2)	44.0 (42.0 45.3)	0.7058
GLB (g/L)	28.02 ± 3.75	28.48 ± 3.87	0.4336
TBIL (mol/L)	15.2 (12.3 19.)	15.8 (12.5 19.0)	0.4985
DBIL (mol/L)	5.2 (3.9 6.3)	5.3 (4.2 6.6)	0.4765
IBIL (mol/L)	10.1 (7.9 12.4)	10.3 (7.8 13.2)	0.7194
NAR	0.092 (0.078 0.116)	0.086 (0.071 0.111)	0.1363
NTBR	0.279 (0.195 0.371)	0.215 (0.168 0.322)	0.1043
NIBR	0.421 (0.280 0.572)	0.342 (0.248 0.495)	0.1254

Data are presented as median (IQR) except for GLB, which is presented as mean ± SD. Kolmogorov-Smirnov test was performed for checking data normality. The differences of all parameters between patients with PACG and POAG were examined by Mann–Whitney test, except for disease severity and GLB, which was examined by Chi-square test and unpaired Student's *t* test (two-tailed), respectively. *p* < 0.05 was considered statistically significant.

**Table 3:** The discriminative abilities of variables between patients with glaucoma and healthy controls.

	Glaucoma vs. healthy controls		
	AUC	*p*	Max. Youden
ALB	0.6253	<0.0001	0.22
TBIL	0.5772	0.0052	0.16
IBIL	0.6105	<0.0001	0.22
NAR	0.6673	<0.0001	0.25
NTBR	0.6338	<0.0001	0.23
NIBR	0.6566	<0.0001	0.27

Receiver operating characteristic (ROC) curve analysis was performed. Youden index was calculated as sensitivity + specificity‐1. *p* < 0.05 was considered statistically significant. Abbreviations: AUC: area under the ROC curve; Max. Youden: the Youden index maximum.

**Table 4:** The discriminative abilities of variables in patients with glaucoma, stratified according to disease severity.

	Early vs. moderate			Early vs. severe			Moderate vs. severe		
	AUC	*p*	Max. Youden	AUC	*p*	Max. Youden	AUC	*p*	Max. Youden
ALB	0.5356	0.5377	0.09	0.6096	0.0553	0.21	0.6353	0.0069	0.22
IBIL	0.5899	0.1203	0.27	0.6345	0.0187	0.30	0.5190	0.7051	0.13
NAR	0.6672	0.0039	0.30	0.7554	<0.0001	0.46	0.6040	0.0378	0.25
NTBR	0.6713	0.0031	0.34	0.7027	0.0004	0.36	0.5177	0.7234	0.08
NIBR	0.6934	0.0008	0.35	0.7250	<0.0001	0.40	0.5153	0.7604	0.08

The severity of glaucoma was determined based on the mean deviation (MD) of visual field: early indicates visual field MD of greater than -6 dB; moderate, -12 dB to -6 dB; and severe, no greater than -12 dB. Receiver operating characteristic (ROC) curve analysis was performed. Youden index was calculated as sensitivity + specificity‐1. *p* < 0.05 was considered statistically significant.

## Data Availability

All data used to support the findings of this study are included within the article.

## Abbreviations

PACG: Primary angle-closure glaucoma
POAG: Primary open-angle glaucoma
IOP: Intraocular pressure
MD: Mean deviation
SD: Standard deviation
IQR: Interquartile range
TP: Total protein
ALB: Albumin
GLB: Globulin
A/G: Albumin-to-globulin ratio
TBIL: Total bilirubin
DBIL: Direct bilirubin
IBIL: Indirect bilirubin
NAR: Neutrophil-to-albumin ratio
NTBR: Neutrophil-to-total bilirubin ratio
NIBR: Neutrophil-to-indirect bilirubin ratio
NLR: Neutrophil-to-lymphocyte ratio
NPR: Neutrophil-to-platelet ratio
ROS: Reactive oxygen species
CNS: Central nervous system
ROC: Receiver operating characteristics
AUC: Area under the ROC curve.
