# Correction to “Clinical Significance of Albumin‐ and Bilirubin‐Based Biomarkers in Glaucoma: A Retrospective Case–Control Study”

**DOI:** 10.1155/omcl/9767860

**Published:** 2026-07-01

C. He, G. Zhang, J. Fu, et al., “Clinical Significance of Albumin‐ and Bilirubin‐Based Biomarkers in Glaucoma: A Retrospective Case–Control Study,” *Oxidative Medicine and Cellular Longevity* 2022, no. 1 (2022): 8063651, https://doi.org/10.1155/2022/8063651.

In the above article, errors during manuscript preparation led to the values for “Age” and “Glaucoma Severity” in Table 1 to be incorrect. The correct Table 1 is as follows:

**Table 1:** Demographics and parameters of glaucoma patients and healthy controls.

	Glaucoma	Healthy controls	*p* value
*n*	175	293	—
Age (year)	62 (54–71)	64 (60–74)	0.0007
Gender			
Female	94	136	0.1517
Male	81	157	
PACG/POAG	105/70	—	—
IOP (mmHg)	21.1 (16.8–32.5) ↑	15.8 (12.5–17.8)	<0.0001
Glaucoma severity			
Early	41	—	—
Moderate	65	—	—
Severe	69	—	—
NEU ( ×10^9^/L)	4.0 (3.3–5.0) ↑	3.4 (2.7–4.1)	<0.0001
TP (g/L)	71.9 (68.6–74.7) ↓	72.5 (70.0–75.6)	0.0087
ALB (g/L)	43.5 (42.1–45.3) ↓	44.6 (43.1–46.6)	<0.0001
GLB (g/L)	28.21 ± 3.76	28.22 ± 3.55	0.9770
TBIL (mmol/L)	15.4 (12.3–19.2) ↓	17.2 (13.9–20.7)	0.0052
DBIL (mmol/L)	5.2 (4.1–6.5)	4.9 (4.2–5.9)	0.0925
IBIL (mmol/L)	10.2 (7.9–12.7) ↓	11.8 (9.5–14.9)	<0.0001
NAR	0.092 (0.075–0.116) ↑	0.076 (0.060–0.094)	<0.0001
NTBR	0.247 (0.197–0.354) ↑	0.204 (0.149–0.267)	<0.0001
NIBR	0.382 (0.280–0.552) ↑	0.283 (0.207–0.391)	<0.0001

*Note:* Data are presented as median (IQR) except for GLB, which is presented as mean ± SD. Kolmogorov–Smirnov test was performed for checking data normality. The differences of all parameters between glaucoma patients and healthy controls were examined by Mann–Whitney test, except for the gender and GLB, which was examined by Chi‐square test and unpaired Student’s *t* test (two‐tailed), respectively. *p* < 0.05 was considered statistically significant.

Abbreviations: ALB, albumin; DBIL, direct bilirubin; GLB, globulin; IBIL, indirect bilirubin; IOP, intraocular pressure; IQR, interquartile range; NAR, neutrophil‐to‐albumin ratio; NEU, neutrophil counts; NIBR, neutrophil‐to‐indirect bilirubin ratio; NTBR, neutrophil‐to‐total bilirubin ratio; PACG, primary angle‐closure glaucoma; POAG, primary open‐angle glaucoma; TBIL, total bilirubin; TP, total protein.

In addition, the *y*‐axis labels for Figure 1c,f are incorrect. The correct Figure 1 is as follows:

**Figure 1:**
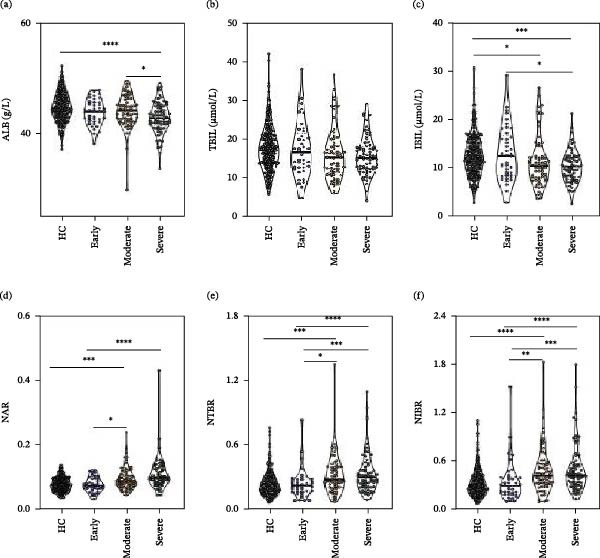
Associations of serum ALB, BIL, NAR, NTBR, and NIBR with disease severity. Serum levels of (a) ALB, (b) TBIL, (c) IBIL, (d)NAR, (e) NTBR, and (f) NIBR among different glaucoma patient groups stratified according to disease severity. The severity of glaucoma was determined based on the mean deviation (MD) of visual field: early (*n* = 41) indicates visual field MD of greater than −6 dB; moderate (*n* = 65), −12 dB to −6 dB; and severe (*n* = 69), no greater than −12 dB. Kolmogorov–Smirnov test was performed for checking data normality. The differences of all parameters among three groups were first examined by Kruskal–Wallis test, followed by Dunn’s multiple comparisons test to determine the differences of each group with every other group (adjusted *p* values are shown).  ^∗^ *p*  < 0.05,  ^∗∗^ *p*  < 0.01,  ^∗∗∗^ *p*  < 0.001, and  ^∗∗∗∗^ *p*  < 0.0001. HC: healthy controls; ALB: albumin; TBIL: total bilirubin; IBIL: indirect bilirubin; NAR: neutrophil‐to‐albumin ratio; NTBR: neutrophil‐to‐total bilirubin ratio; NIBR: neutrophil‐to‐indirect bilirubin ratio.

We apologize for these errors.

